# A Rapid and Cost-Effective Pipeline to Identify and Capture BGCs From Bacterial Draft Genomes

**DOI:** 10.21769/BioProtoc.5549

**Published:** 2025-12-20

**Authors:** Marco A. Campos-Magaña, Vitor A. P. Martins dos Santos, Luis Garcia-Morales

**Affiliations:** 1 Dept. Bioprocess Engineering, Wageningen University and Research, Wageningen, the Netherlands; 2 Dept. Systems and Synthetic Biology, Wageningen University and Research, Wageningen, the Netherlands; 3 Lifeglimmer GmbH, Berlin, Germany

**Keywords:** Biosynthetic gene clusters, Genome mining, Transformation-associated recombination cloning, Myxobacteria, Microbial genomics, Oxford nanopore sequencing, AntiSMASH

## Abstract

The exploration of microbial genomes through next-generation sequencing (NGS) and genome mining has transformed the discovery of natural products, revealing an immense reservoir of previously untapped chemical diversity. Bacteria remain a prolific source of specialized metabolites with potential applications in medicine and biotechnology. Here, we present a protocol to access novel biosynthetic gene clusters (BGCs) that encode natural products from soil bacteria. The protocol uses a combination of Oxford Nanopore Technology (ONT) sequencing, de novo genome assembly, antiSMASH for BGC identification, and transformation-associated recombination (TAR) for cloning the BGCs. We used this protocol to allow the detection of large BGCs at a relatively fast and low-cost DNA sequencing. The protocol can be applied to diverse bacteria, provided that sufficient high-molecular-weight DNA can be obtained for long-read sequencing. Moreover, this protocol enables subsequent cloning of uncharacterized BGCs into a genome engineering-ready vector, illustrating the capabilities of this powerful and cost-effective strategy.

Key features

• This protocol enables bioprospection through cloning of a novel BGC identified in an ONT bacterial draft genome.

• A combination of ONT sequencing, antiSMASH, and TAR cloning can be used to clone BGCs from bacteria into a vector.

• Cost-effective strategy for the discovery of BGCs of diverse natural product classes, including nonribosomal peptides, polyketides, and RiPPs.

• Overnight sequencing in-house using cheap and easy-to-use instruments such as MinION, which allows multiplexing.

## Graphical overview


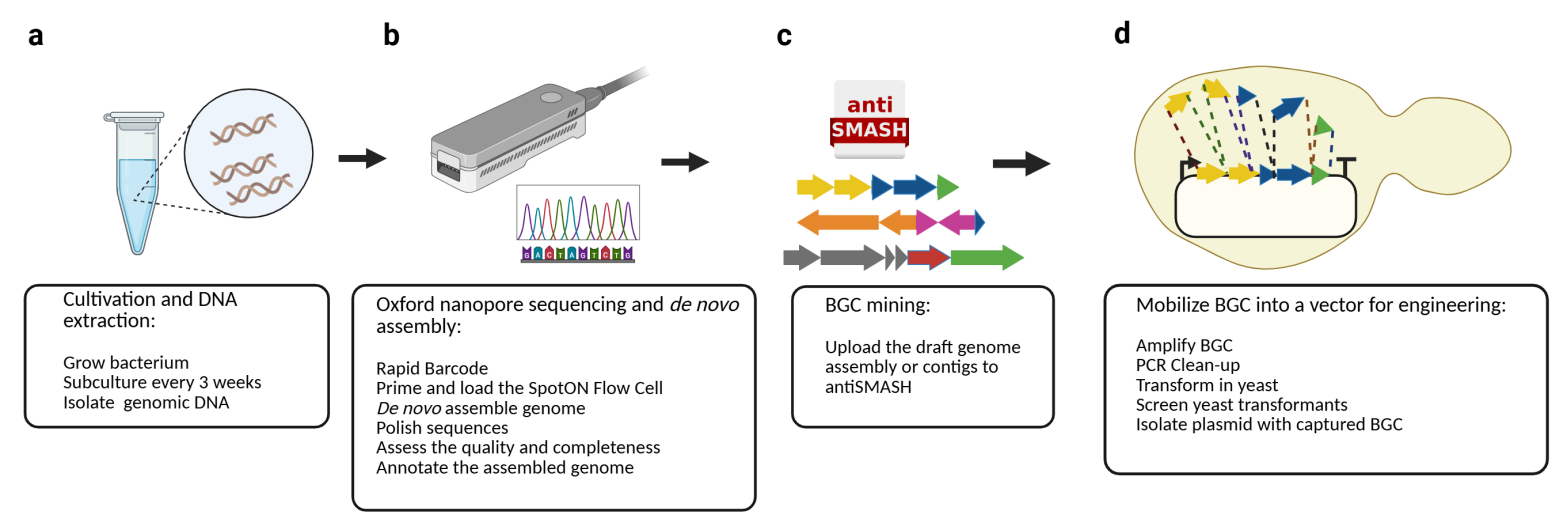


**Overview of the pipeline.** The protocol consists of four main steps: (a) Cultivation and isolation of genomic DNA of the bacterium of interest, (b) Oxford Nanopore sequencing and de novo genome assembly, (c) BGC mining using antiSMASH, and (d) biosynthetic gene cluster (BGC) mobilization into a genome engineering-ready vector via PCR amplification and TAR cloning in yeast.

## Background

Microbial natural products are chemically diverse secondary metabolites with ecological functions and major applications in drug discovery and biotechnology. Many of these compounds are produced by soil and marine bacteria, yet their exploitation is often hampered by difficulties in cultivation and expression under laboratory conditions [1]. A key advance in natural product research has been the identification of biosynthetic gene clusters (BGCs), which encode the enzymatic machinery for metabolite production, regulation, transport, and, if necessary, resistance to the BGC metabolite. Genome sequencing and mining have accelerated the discovery of BGCs that encode enzymes for the production of secondary metabolites. However, there is still a limited number of genomes available from bacteria rich in secondary metabolites, such as myxo- and actinobacteria [2] in comparison to bacterial genome sequences of human pathogens.

The discovery of novel natural products increasingly relies on the systematic mining of BGCs. While many bacteria, such as myxobacteria and actinobacteria, are renowned for their richness in biosynthetic potential, their limited amenability to genetic manipulation makes direct engineering a major challenge. As an alternative, heterologous expression of BGCs in well-characterized laboratory microorganisms has emerged as a promising strategy [3–7]. However, this approach involves overcoming several technical bottlenecks: from sequencing and identifying large, GC-rich, and repetitive genomes, to cloning and refactoring BGCs, introducing them into suitable hosts, achieving heterologous production, and finally isolating and characterizing the resulting compounds. Recent technological advances have addressed many of these hurdles. For instance, genome mining tools such as antiSMASH have streamlined BGC identification [8], while CRISPR-based approaches have allowed the refactoring of large gene clusters [9,10]. Similarly, versatile genome engineering platforms such as chassis-independent recombinase-assisted genome engineering (CRAGE) facilitate transfer into diverse bacterial hosts [11]. Current heterologous expression strategies vary in their reliance on genome sequence quality: while de novo DNA synthesis requires highly accurate BGC sequences [12–14], methods such as transformation-associated recombination (TAR) cloning [15,16] and Cas9-assisted targeting of chromosome segments (CATCH) [17] are compatible with draft genomes, depending on oligonucleotides for primer or gRNA design. Draft genome sequencing itself has become increasingly accessible with ONT long-read platforms due to reduced costs and multiplexing capability. Generating highly accurate assemblies of large, repetitive, GC-rich genomes still requires combined short- and long-read approaches, which remain costly when scaled to numerous strains. However, draft genome sequencing allows the detection and cloning of a bacterial BGC [18]. Several technologies for whole genome sequencing exist, including Illumina, PacBio, and ONT. While PacBio sequencing offers higher accuracy, ONT provides a more affordable and scalable option, delivering data of sufficient quality for BGC cloning, as shown in Campos-Magaña et al. [18]. Alternatively, hybrid ONT/Illumina strategies can be employed to generate high-quality genome assemblies [19]. However, currently, the cost of a hybrid approach is more expensive than sequencing only using ONT. Furthermore, ONT sequencing prices can be even lowered by performing it in-house using cheap and easy-to-use instruments such as MinION, which allows multiplexing. This protocol enables the generation of a draft genome using ONT sequencing and extracted bacterial genomic DNA. This protocol combines ONT sequencing with antiSMASH and TAR cloning for scalable discovery and capture of BGCs. We used TAR cloning in yeast due to its ability to clone large constructs via in vivo homologous recombination, allowing us to precisely clone BGCs identified in Oxford Nanopore draft genomes, despite the inherent limitations and reduced accuracy of this sequencing platform. This strategy is also compatible with subsequent refactoring of captured BGCs, an important step that involves replacing native regulatory elements with more suitable ones for heterologous expression, since the final vector with the captured BGC can serve as input DNA for multiplex editing using CRISPR-Cas9 [20–22] and mobilization into diverse heterologous hosts using CRAGE [11]. Such a streamlined, rapid, and low-cost approach applied to the large collections of non-sequenced bacteria will enable exploration of BGCs in microbes.

## Materials and reagents

**Biological materials**

1. ATCC *Saccharomyces cerevisiae*; W303-1a (ATCC, 50-238-3847)

2. *Aetherobacter fasciculatus* SBSr002 (Leibniz-Institute DSMZ-German Collection of Microorganisms and Cell Cultures, DSM 24601)

3. TransforMax EPI300 Electrocompetent *E. coli* (Epicentre^TM^, EC300110)

4. pCC1FOS (Epicentre Technologies, V008674)

5. pRS314 (ATCC, Plasmid #77143)

6. pRS315 (ATCC, Plasmid #77144)

7. pk18mobsacB (ATCC, Plasmid #87097)

8. *E. coli* DH5α competent cells (ThermoFisher Scientific, catalog number: 18265017)

**Reagents**

1. GenElute^TM^ Bacterial Genomic DNA kit (Sigma-Aldrich, catalog number: NA2120)

2. Agentcourt AMPure XP beads (Beckman Coulter, catalog number: A63880)

3. Rapid Barcoding kit (Oxford Nanopore Technologies, catalog number: SQK-RBK004)

4. Q5^®^ high-fidelity DNA polymerase (New England BioLabs, catalog number: M0491S)

5. Phire^TM^ Hot Start II DNA polymerase (Thermo Scientific, catalog number: F124S)

6. β-Mercaptoethanol (Millipore, catalog number: 444203)

7. Zymolyase-100T (Carl Roth, catalog number: 9329.1)

8. Vitamin B12 (Sigma-Aldrich, catalog number: V2876)

9. Yeast extract (Gibco, catalog number: 211931)

10. CaCl_2_ × 2H_2_O (Sigma-Aldrich, catalog number: 223506)

11. Agar (Difco, catalog number: 214530)

12. Sorbitol (Millipore, catalog number: 56755-M)

13. EDTA (Sigma-Aldrich, catalog number: EDS-100G)

14. Bacto peptone (Gibco, catalog number: DF0118-17-0)

15. Tris HCl (Roche, catalog number: 10812846001)

16. Na_2_HPO_4_ × 2H_2_O (Sigma-Aldrich, catalog number: 71643)

17. NaH_2_PO_4_ × 2H_2_O (Sigma-Aldrich, catalog number: 71505)

18. PEG 8000 (Fisher Scientific, catalog number: BP233-1)

19. Minimal SD base (Takara, catalog number: 630411)

20. Yeast synthetic drop-out medium supplement, without leucin, DO-Leu (Takara, catalog number: 630414)

21. NaOH (Supelco, catalog number: 1091401,000)

22. Sodium dodecyl sulfate (Sigma-Aldrich, catalog number: 436143)

23. YPDA (Takara, catalog number: 630464)

24. Yeast DNA Extraction kit (Thermo Scientific, catalog number: 78870)

25. 1 kb Plus DNA ladder (New England Biolabs, catalog number: N3200S)

26. Gel loading dye, purple (New England Biolabs, catalog number: B7025)

27. Gentamycin sulfate salt (BioReagent, catalog number: G1264)

28. Glycerol (Sigma-Aldrich, catalog number: 49767)

29. Chloramphenicol (Sigma-Aldrich, catalog number: C0378)

30. Buffer TAE (Sigma-Aldrich, catalog number: 574797)

31. KOH (Sigma-Aldrich, catalog number: P1767)

32. Oligonucleotides for amplification and colony PCR primers (Integrated DNA Technologies), storage: -20 °C

33. MIDORI Green Easy (Nippon genetics, catalog number: MG12)

34. NucleoSpin Gel and PCR Clean-up (Macherey-Nagel, catalog number: 740609.50)

35. GeneJET Plasmid Miniprep kit (Thermo Scientific, catalog number: K0502)

36. CHEF Genomic DNA Plug kits (Bio-Rad, catalog number: 170-3592)

37. HpaI (New England Biolabs, catalog number: R0105S)

38. BamHI-HF (New England Biolabs, catalog number: R3136S)

39. NaCl (Sigma-Aldrich, catalog number: S9625)

40. Tryptone (OXOID, catalog number: LP0042)

41. Baker's yeast (Bruggeman, catalog number: 2001-0580)

**Solutions**

1. VY/2 media (see Recipes)

2. EDTA 0.5 M pH 7.5 (see Recipes)

3. Sorbitol 1 M (see Recipes)

4. SOS (see Recipes)

5. Tris-HCl 1 M pH 7.5 (see Recipes)

6. SPEM pH 7.5 (see Recipes)

7. STC (see Recipes)

8. PEG (see Recipes)

9. Zymolyase stock (see Recipes)

10. TOP agar-leu (or -Trp) (see Recipes)

11. Sorb plates -Leu (or -Trp) (see Recipes)

12. Liquid media YPDA (see Recipes)

13. SDS (see Recipes)

14. Reaction setup for Q5^®^ High-Fidelity DNA Polymerase (see Recipes)

15. Reaction setup for Phire^TM^ Hot Start II DNA Polymerase (see Recipes)

16. Gentamycin (see Recipes)

17. CaCl_2 _1 M (see Recipes)

18. Golden Gate assembly mixture (see Recipes)

19. Chloramphenicol (see Recipes)

20. KOH (see Recipes)

21. Glycerol (see Recipes)

22. LB medium (see Recipes)

**Recipes**

**1. VY/2 media**

**Table:** 

Reagent	Final concentration	Quantity or volume
Baker’s yeast	5 g/L	5 g
CaCl_2_ × 2H_2_O	1.36 g/L	1.36 g
Vitamin B12	0.5 mg/L	0.5 mg
Agar (Difco)	15 g/L	15 g
Distilled water	n/a	1,000 mL

*Note: Autoclave. Adjust to pH 7.2 with KOH.*

**2. EDTA 0.5 M pH 7.5**

**Table:** 

Reagent	Final concentration	Quantity or volume
CaCl_2_	0.18612 g/mL	46.53 g
Distilled water	n/a	250 mL

*Note: Filter sterilize the solution using a 0.22 μm sterile filter.*

**3. Sorbitol 1 M**

**Table:** 

Reagent	Final concentration	Quantity or volume
Sorbitol	182 g/L	182 g
Distilled water	n/a	1,000 mL

*Note: Filter sterilize the solution using a 0.22 μm sterile filter. Storage: 6 months at room temperature (RT).*

**4. SOS**

**Table:** 

Reagent	Final concentration	Quantity or volume
Sorbitol	0.182 g/mL	9.1 g
Yeast extract	0.0025 g/mL	0.125 g
Bacto peptone	0.005 g/mL	0.25 g
CaCl_2_	6.5 mM	300 μL
Distilled water	n/a	50 mL

*Note: Filter sterilize the solution using a 0.2 μm sterile filter. Store for 6 months at RT. Aliquot in 5 mL volumes.*

**5. Tris-HCl 1 M pH 7.5**

**Table:** 

Reagent	Final concentration	Quantity or volume
Tris-HCl 1 M pH 7.5	121.4 g/L	121.4 g
Distilled water	n/a	1,000 mL

*Note: Filter sterilize the solution using a 0.2 μm sterile filter.*

**6. SPEM pH 7.5**

**Table:** 

Reagent	Final concentration	Quantity or volume
Na_2_HPO_4_ × 2H_2_O	0.00138 g/mL	0.69 g
NaH_2_PO4 × 2H_2_O	0.00036 g/mL	0.18 g
EDTA pH 7.5	0.5 M	10 mL
Sorbitol 1 M	0.182 g/mL	91 g
Distilled water	n/a	500 mL

*Note: Filter sterilize the solution using a 0.2 μm sterile filter. Store for 6 months at RT. Aliquot in 10 mL volumes.*

**7. STC**

**Table:** 

Reagent	Final concentration	Quantity or volume
Sorbitol	0.182 g/mL	18.2 g
Tris-HCl 1 M pH 7.5	0.01 M	1 mL
CaCl_2_ 1 M	0.01 M	1 mL
Distilled water	n/a	100 mL

*Note: Filter sterilize the solution using a 0.2 μm sterile filter. Store for 6 months at RT. Aliquot in 1 mL volumes.*

**8. PEG**

**Table:** 

Reagent	Final concentration	Quantity or volume
PEG 8000	20%	4 g
Tris-HCl 1 M pH 7.5	10 mM	200 μL
CaCl_2_ 1 M	10 mM	200 μL
Distilled water	n/a	20 mL

*Note: Heat mildly if necessary. Filter sterilize the solution using a 0.2 μm sterile filter. Prepare fresh.*

**9. Zymolyase stock**

**Table:** 

Reagent	Final concentration	Quantity or volume
Zymolyase-100T	10 mg/mL	200 mg
Tris-HCl 1 M pH 7.5	0.1 M	1 mL
Glycerol 50%	5%	10 mL
Distilled water	n/a	9 mL

*Note: Aliquot and store for 6 months at -20 °C.*

**10. TOP agar-leu (or -Trp)**

**Table:** 

Reagent	Final concentration	Quantity or volume
Sorbitol 1 M	0.182 g/mL	91 g
SD base	0.0267 g/mL	13.35 g
DO-Leu (or -Trp)	0.0007 g/mL	0.35 g
Agar	0.03 g/mL	15 g
Distilled water	n/a	500 mL

*Note: Autoclave and store at 65 °C. Adjust to pH 5.8 with NaOH 1 M.*

**11. Sorb plates -Leu (or -Trp)**

**Table:** 

Reagent	Final concentration	Quantity or volume
Sorbitol	1 M	145.6 g
SD base	0.0267 g/mL	21.36 g
DO-Leu (or -Trp)	0.0006875 g/mL	0.55 g
Agar	0.02 g/mL	16 g
Distilled water	n/a	800 mL

*Note: Autoclave and store the Sorb agar bottle at 65 °C. Adjust to pH 5.8 with NaOH 1 M.*

**12. Liquid media YPDA**

**Table:** 

Reagent	Final concentration	Quantity or volume
YPDA	1×	25 g
Distilled water	n/a	500 mL

*Note: Autoclave.*

**13. SDS**

**Table:** 

Reagent	Final concentration	Quantity or volume
SDS	0.02 g/mL	2 g
Distilled water	n/a	100 mL

**14. Reaction setup for Q5^®^ High-Fidelity DNA Polymerase**

**Table:** 

Reagent	Final concentration	Quantity or volume for 25 μL reaction
5× Q5^®^ reaction buffer	1×	5 μL
10 mM dNTPs	200 μM	0.5 μL
10 μM forward primer	0.5 μM	1.25 μL
10 μM reverse primer	0.5 μM	1.25 μL
Template DNA	<1,000 ng	Variable (usually 0.25 µL)
Q5^®^ High-Fidelity DNA Polymerase	0.02 U/µL	0.25 μL
5× Q5^®^ High GC Enhancer	1×	5 μL
Nuclease-free water	-	to 25 μL

**15. Reaction setup for Phire^TM^ Hot Start II DNA Polymerase**

**Table:** 

Reagent	Final concentration	Quantity or volume for 20 μL reaction
5× Phire Green reaction buffer	1×	4 μL
10 mM dNTPs	200 μM	0.4 μL
10 μM forward primer	0.5 μM	1 μL
10 μM reverse primer	0.5 μM	1 μL
Template DNA	-	Variable
Phire^TM^ Hot Start II DNA Polymerase	-	0.4 μL
DMSO	1×	0.6 μL
Nuclease-free water	-	Add to 20 μL

**16. Gentamycin**

**Table:** 

Reagent	Final concentration	Quantity or volume
Gentamycin sulfate salt	15 μg/mL	150 μg
Distilled water	n/a	10 mL

*Note: Filter sterilize the solution using a 0.2 μm sterile filter. Store at -20 °C. Aliquot in 1 mL volumes.*

**17. CaCl_2 _1 M**

**Table:** 

Reagent	Final concentration	Quantity or volume
CaCl_2_	0.111 g/mL	1.11 g
Distilled water	n/a	10 mL

*Note: Filter sterilize the solution using a 0.2 μm sterile filter.*

**18. Golden Gate assembly mixture**

**Table:** 

Reagent	Final concentration	Quantity or volume
BsaI-HF^®^ v2		10 μL
T4 DNA ligase		10 μL
T4 buffer		15 μL
BSA		1.5 μL
MilliQ water		13.5 μL

**19. Chloramphenicol**

**Table:** 

Reagent	Final concentration	Quantity or volume
Chloramphenicol	35 μg/mL	500 mg
95% ethanol	n/a	14.28 mL

*Note: Filter sterilize the solution using a 0.2 μm sterile filter. Store at -20 °C. Aliquot in 1 mL volumes.*

**20. KOH**

**Table:** 

Reagent	Final concentration	Quantity or volume
Potassium hydroxide	0.1 M	5.6 g
Distilled water	n/a	1,000 mL

*Note: Filter sterilize the solution using a 0.2 μm sterile filter. Store at -20 °C. Aliquot in 1 mL volumes.*

**21. Glycerol**

**Table:** 

Reagent	Final concentration	Quantity or volume
Glycerol	50%	25 mL
Distilled water	n/a	25 mL

*Note: Autoclave.*

**22. LB medium**

**Table:** 

Reagent	Final concentration	Quantity or volume
Tryptone	10 g/L	10 g
NaCl	10 g/L	10 g
Yeast extract	5 g/L	5 g
Distilled water	n/a	1,000 mL

*Note: Autoclave.*

**Laboratory supplies**

1. Vented cap Greiner tubes (Greiner bio-one, catalog number: 115262)

2. UV cuvettes (Brand, catalog number: Z628026)

3. 10 μL pipette tips (Brand 732724, catalog number: Z740051)

4. 20 μL pipette tips (Brand 732724, catalog number: Z740051)

5. 200 μL pipette tips (Brand 732724, catalog number: Z740051)

6. 1,000 μL pipette tips (Brand 732724, catalog number: Z740051)

7. Erlenmeyer flasks 500 mL (Duran, catalog number: 212174405)

8. 1.5 mL microcentrifuge tube (Brand, catalog number: 525-0944)

9. Falcon tubes 50 mL (Corning, catalog number: 352098)

10. 0.2 mL thin-walled PCR tubes (VWR, catalog number: 732-1521)

11. Gene Pulser/MicroPulser electroporation cuvettes, 0.2 cm gap (Bio-Rad, catalog number: 1652082)

12. 0.22 μm syringe filter (Millipore, catalog number: SLGPR33RS)

13. Syringe PP/PE without needle (Sigma-Aldrich, catalog number: Z683604)

## Equipment

1. Microfuge (Benchmark Scientific C1008-R-E-UK, catalog number: Z764205)

2. Microcentrifuge (Eppendorf, catalog number: 5406000119)

3. Pipette 2–20 μL (Thermofisher, catalog number: 4642060)

4. Pipette 20–200 μL (Thermofisher, catalog number: 4642080)

5. Pipette 100–1,000 μL (Thermofisher, catalog number: 4642090)

6. Freezer (-20 °C)

7. Refrigerator (2–8 °C)

8. Benchtop centrifuge (Thermo Scientific, catalog number: 28523)

9. Incubator (FED, catalog number: 260)

10. Incubator shaker (New Brunswick Innova^®^, catalog number: M1335-0002)

11. Thermomixer (Eppendorf, catalog number: EP5387000030)

12. OD meter 600 nm (Implen Diluphotometer^TM^, catalog number: C40)

13. Nanodrop (Thermofisher, catalog number: ND-2000)

14. MinION Flow Cell R9.4.1 (Oxford Nanopore Technologies, catalog number: FLO-MIN106D)

15. MinION (Oxford Nanopore Technologies, M1DCapEx)

16. Electronic Pipette Controller (Eppendorf^®^ Easypet^®^, catalog number: EP4430000018)

17. Fume hood

18. Laminar flow hood

19. Mupid-One Electrophoresis Unit (MUPIDONE, catalog number: MU2)

20. Gel casting tray (MUPIDONE, catalog number: ON-MS)

21. Well combs (MUPIDONE, catalog number: AC-C1)

22. Mastercycler Nexus X2 Thermal Cyclers Nexus GX2e satellite unit (Eppendorf, catalog number: EP6338000012)

23. Gel Doc XR+ (Bio-Rad, catalog number: 170-8195)

24. Gene Pulser Xcell Electroporation Systems (Bio-Rad, catalog number: 1652660)

25. pH meter (Mettler Toledo, catalog number: MT30266626)

26. Hula mixer (rotator mixer) (Thermo Scientific, catalog number: 15920D)

## Software and datasets

1. Guppy Oxford Nanopore Technologies 4.4.1(https://github.com/nanoporetech/pyguppyclient)

2. Flye genome assembler v2.9.5 (https://github.com/mikolmogorov/Flye)

3. Medaka polisher v1.12.0 (https://github.com/nanoporetech/medaka).

4. antiSMASH v7.1 (https://github.com/antismash/antismash/releases)

5. checkM v1.2.3 (https://github.com/Ecogenomics/CheckM)

6. BUSCO 5.6.0 (https://gitlab.com/ezlab/busco)

7. Bakta v1.9.3 (https://github.com/oschwengers/bakta).

8. COG classifier tool v1.0.5 (https://github.com/moshi4/COGclassifier).

All data have been deposited in the European Nucleotide Archive under the accession PRJEB72099 and Campos-Magaña et al. [18].

## Procedure

**A. Isolation of genomic DNA**

1. For this protocol, we selected the myxobacterium *A. fasciculatus SBSr002*, which grows in VY/2 media with an incubation period of 8–14 days. Grow this myxobacterium in 10 mL of VY/2 media at 28 °C in a liquid culture (200–250 rpm, vented cap Greiner tubes).

*Note: Subculture (at least) every three weeks due to their slow growth. Change the liquid medium weekly with 10 mL of fresh medium. If there is no growth after 10 days, carefully split up the agar culture cubes, squeeze the material to the agar plate, and reincubate*.

2. Isolate genomic DNA from 10 mL cultures of actively growing (exponential/mid-log) cultures of *A. fasciculatus* SBSr002, using the GenElute^TM^ Bacterial Genomic DNA kit for Gram-negative, following the manufacturer’s instructions. Elute the DNA in Tris-EDTA solution. Prepare agarose plugs containing genomic DNA from this strain according to the protocol “Preparation of Agarose Embedded Bacterial DNA” by Bio-Rad (CHEF Genomic DNA Plug Kits) found at https://www.bio-rad.com/webroot/web/pdf/lsr/literature/LIT510.pdf. Prepare all plugs with 1% agarose and four different culture concentrations (1:1 with 60 μL of culture, 1:2 with 30 μL of culture, 1:4 with 15 μL of culture, and 1:8 with 7.5 μL of culture).

3. Use Agentcourt AMPure XP beads to concentrate and purify 100 μL of genomic DNA down to 10 μL, with 180 μL of beads (1.8× proportion) following the manufacturer’s instructions.

**B. Rapid barcoding sequencing (SQK-RBK004)**

*Note: Before your first run, we recommend all new users watch the “Rapid Barcoding Sequencing (SQK-RBK004)” barcoding image found at* *https://nanoporetech.com/document/rapid-barcoding-sequencing-sqk-rbk004* . *All steps in this section are performed at room temperature (RT).*

1. Thaw Rapid Barcoding kit components at RT, spin down briefly using a microfuge, and mix by pipetting in the order indicated below:

a. Fragmentation mix RB01-12: not frozen, briefly spin down, mix well by pipetting.

b. Rapid adapter (RAP): not frozen, briefly spin down, mix well by pipetting.

c. Sequencing buffer (SQB): thaw at RT, briefly spin down, mix well by pipetting*.

d. Loading beads (LB): thaw at RT, briefly spin down, mix by pipetting or vortexing immediately before use.

e. Flush buffer (FLB)-1 tube: thaw at RT, briefly spin down, mix well by pipetting*.

f. Flush tether (FLT): thaw at RT, briefly spin down, mix well by pipetting.

*Note: Vortexing, followed by a brief spin in a microfuge, is recommended for SQB and FLB.*

2. Resuspend the DNA in nuclease-free water.

3. Transfer ~400 ng of genomic DNA into a DNA LoBind tube.

4. Adjust the volume to 7.5 μL with nuclease-free water.

5. Mix by finger flicking the tube to avoid unwanted shearing.

6. Spin down briefly in a microfuge.

7. In a 0.2 mL thin-walled PCR tube, mix the following:

a. 7.5 μL of 400 ng of template DNA.

b. 2.5 μL of fragmentation mix RB01-12 (one for each sample).

8. Mix gently by finger flicking the tube and spin down.

9. Incubate the tube at 30 °C for 1 min and then at 80 °C for 1 min. Briefly (2 min), put the tube on ice to cool it down.

10. Pool the barcoded samples in a 1.5 mL Eppendorf DNA LoBind tube. The expected volume is ~10 μL per sample.

11. Resuspend the AMPure XP beads by vortexing.

12. To the entire pooled barcoded sample from step B10, add an equal volume of resuspended AMPure XP beads and mix by flicking the tube.

13. Incubate on a Hula mixer (rotator mixer) for 5 min at RT.

14. Prepare 500 μL of fresh 70% ethanol in nuclease-free water.

15. Spin down the sample and pellet on a magnet. Keep the tube on the magnet and pipette off the supernatant.

16. Keep the tube on the magnet and wash the beads with 200 μL of freshly prepared 70% ethanol without disturbing the pellet. Remove the ethanol using a pipette and discard.

17. Repeat the previous step.

18. Spin down and place the tube back on the magnet. Pipette off any residual 70% ethanol. Allow to dry for 5 min at RT.

19. Remove the tube from the magnetic rack and resuspend the pellet in 10 μL of 10 mM Tris-HCl, pH 7.5–8.0, with 50 mM NaCl. Incubate for 2 min at RT.

20. Pellet the beads on a magnet until the eluate is clear and colorless, for at least 1 min.

21. Remove and retain 10 μL of eluate into a clean 1.5 mL Eppendorf DNA LoBind tube.

*Note: Dispose of the pelleted beads.*

22. Add 1 μL of RAP to 10 μL of barcoded DNA.

23. Mix gently by finger flicking the tube and spin down.

24. Incubate the reaction for 5 min at RT.

*Note: The prepared library is used for loading into the MinION flow cell. Store the library on ice until ready to load.*

**C. Priming and loading the SpotON flow cell**

*Notes:*

*1. We recommend that all new users watch the “Priming and loading your flow cell” video before their first run; this video can be found at* *https://community.nanoporetech.com/nanopore_learning/lessons/priming-and-loading-your-flow-cell* .

*2. The sequencing tether (SQT) tube will NOT be used in this protocol. It is provided in the kit for potential future product compatibility.*

1. Thaw the SQB, LB, FLT and one tube of FB at RT.

2. Mix the SQB, FLT, and FB tubes by vortexing (each tube on its own) and spin down at RT.

3. Open the MinION Mk1B lid and slide the flow cell under the clip.

4. Slide the priming port cover clockwise to open the priming port.

*Note: Take care when drawing back the buffer from the flow cell. Do not remove more than 20–30 μL and make sure that the array of pores is covered by buffer at all times. Introducing air bubbles into the array can irreversibly damage pores.*

5. After opening the priming port, check for a small air bubble under the cover. Draw back a small volume to remove any bubbles (a few microliters):

a. Set a P1,000 pipette to 200 μL.

b. Insert the tip into the priming port.

c. Turn the wheel until the dial shows 220–230 μL or until you can see a small volume of buffer entering the pipette tip. *Note: Visually check that there is a continuous buffer from the priming port across the sensor array. See a video of priming and loading your flow cells at* *https://community.nanoporetech.com/nanopore_learning/lessons/priming-and-loading-your-flow-cell* .

6. To prepare the flow cell priming mix, add 30 μL of thawed and mixed FLT directly to the tube of thawed and mixed FB and mix by vortexing at RT.

7. Load 800 μL of the priming mix into the flow cell via the priming port, avoiding the introduction of air bubbles. Wait for 5 min. During this time, prepare the library for loading by following the steps below.

8. Thoroughly mix the contents of the LB tubes by vortexing.

9. In a new tube, prepare the library for loading as follows:

a. 34 μL of SQB.

b. 25.5 μL of LB, mixed immediately before use.

c. 4.5 μL of nuclease-free water.

d. 11 μL of DNA library.

*Note: The LB tube contains a suspension of beads. These beads settle very quickly. It is vital that they are mixed immediately before use.*

10. Complete the flow cell priming:

a. Gently lift the SpotON sample port cover to make the SpotON sample port accessible.

b. Load 200 μL of the priming mix into the flow cell via the priming port (not the SpotON sample port), avoiding the introduction of air bubbles.

11. Mix the prepared library gently by pipetting up and down just prior to loading.

12. Add 75 μL of sample to the flow cell via the SpotON sample port in a dropwise fashion. Ensure each drop flows into the port before adding the next.

13. Gently replace the SpotON sample port cover, making sure the bung enters the SpotON port, and close the priming port.

*Note: Install the light shield on your flow cell as soon as the library has been loaded for optimal sequencing output. We recommend leaving the light shield on the flow cell when the library is loaded, including during any washing and reloading steps. The shield can be removed when the library has been removed from the flow cell.*

14. Place the light shield onto the flow cell by carefully placing the leading edge of the light shield against the clip.

*Notes:*

*1. Do not force the light shield underneath the clip. Gently lower the light shield onto the flow cell. The light shield should sit around the SpotON cover, covering the entire top section of the flow cell.*

*2. The MinION flow cell light shield is not secured to the flow cell; careful handling is required after installation.*

15. Close the device lid and set up a sequencing run on MinKNOW.

16. Run the sequencing experiment configured to store Fast5 raw data files by the ONT MinION during sequencing.

**D. Genome assembly**

1. Base-call, demultiplex, and trim the Fast5 raw data files using the data processing toolkit Guppy version 4.4.1 https://github.com/nanoporetech/pyguppyclient. Use the following commands:

guppy_basecaller --input_path “input folder” --save_path “output folder” g --barcode_kits SQK-RBK004 --trim_barcodes

2. Assemble the ONT data into contigs using Flye genome assembler v2.9.5 (https://github.com/mikolmogorov/Flye) [23]. Use the following commands:

Flye --nano-raw “inputfile.fastq” --out-dir “output folder”

3. Polish or correct base-calling errors in the sequences generated by ONT using Medaka polisher v1.12.0 (https://github.com/nanoporetech/medaka). Use the following commands:

medaka_consensus -i “basecalled sample” -d “assembled fastq file” -o output folder

**Figure 1.**
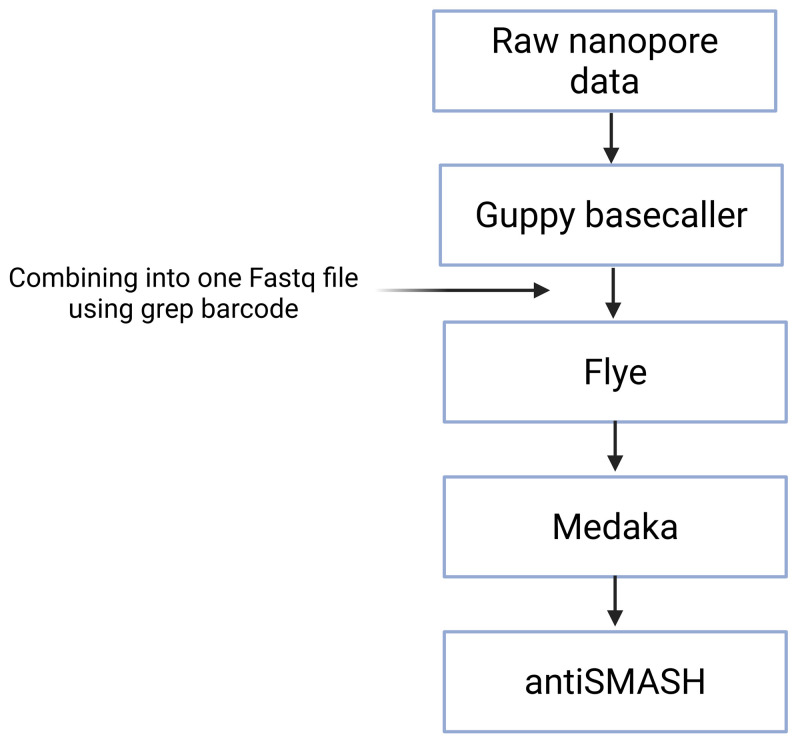
Pipeline from the raw data to identification of biosynthetic gene clusters (BGCs). Fast5 raw data files generated by the Oxford Nanopore Technology (ONT) MinION are base-called, demultiplexed, and trimmed using the data processing toolkit Guppy. Grep barcode is used to locate which reads contain a specific barcode. Flye genome assembler is used to assemble the ONT data into contigs. Medaka polisher is used to polish or correct base-calling errors in the sequences generated by ONT. The draft genome assembly or contigs are uploaded to antiSMASH.

4. Upload the draft genome assembly or contigs to antiSMASH 7.1 (https://github.com/antismash/antismash/releases) [8] with the default strictness (relaxed) of detection. A relaxed strictness means that the tool detects well-defined clusters containing all required parts and partial clusters missing one or more functional parts. This version of antiSMASH includes an RRE-containing detection rule that enables the identification of potentially novel RiPPs clusters. In addition, all extra features during the analysis are selected. The flowchart of the computational pipeline can be found in Figure 1. Use the following commands:

run_antismash input.fasta outputfolder --genefinding-tool prodigal --cb-general --cb-knownclusters --cb-subclusters --asf --pfam2go --smcog-trees

5. Assess the quality and completeness of the assembled genome using checkM v1.2.3 (https://github.com/Ecogenomics/CheckM) [24] and BUSCO v5.6.0 (https://gitlab.com/ezlab/busco) [25].

*Note: CheckM commands used:*

*checkm lineage_wf checkm qa checkm lineage*

*BUSCO commands used:*

*busco -i <input.fasta> -o <output_name> -l <lineage_dataset> -m genome*

6. Upload individual BGCs to Pfam v33.1 [26] and BLAST v2.16.0 [27] to predict the function of the closest homolog based on similarity between sequences. BLAST was used in the blastn mode for query (nucleotide) and database (nucleotide) search.

*Note: Pfam was used under the default settings.*

7. Annotate the assembled genome using Bakta v1.9.3 (https://github.com/oschwengers/bakta) [28].

*Note: BAKTA commands used:*

*bakta --db /path/to/bakta_db --output bakta_out genome.fasta*

8. Classify gene functions into categories using the COG classifier tool v1.0.5 (https://github.com/moshi4/COGclassifier) [29].

*Note: COGclassifier commands used:*

*COGclassifier -i <input.fasta> -o <output_name>*

**E. PCR amplification, digestion, and ligation steps prior to TAR cloning**

1. Amplify the oriT element from the plasmid pk18mobsacB with primers 1–2 (Table S1). See thermocycling conditions in Table 1.

2. Digest the resulting amplicon and the plasmid pCC1FOS with CutSmart^®^ Buffer and the following restriction enzymes (New England Biolabs): HpaI and BamHI-HF. Follow the manufacturer’s instructions for digestion.

*Note: Use 1 µg of DNA (plasmid and insert), with an initial enzyme-to-DNA ratio of 5–10 units of enzyme per µg of DNA. The insert-to-plasmid ratio is typically 1:1 to 3:1 for ligation.*

3. Purify digested DNA. Follow the manufacturer’s instructions of the NucleoSpin Gel and PCR Clean-up kit for purification.

4. Ligate pCC1FOS with oriT to generate pCC1FOS-oriT with T4 DNA ligase and T4 DNA ligase reaction buffer for 1 h at RT. Follow the manufacturer’s instructions for ligation.

*Note: The insert-to-plasmid ratio is typically 1:1 to 3:1 for ligation.*

5. Transform each ligation (with a total volume of 10 μL) into *E. coli* DH5α competent cells using the following steps:

a. Thaw the cells on ice.

b. Quickly add 10 μL of the ligation to the competent cells, next to the flame, mix gently, and incubate on ice for 30 min.

c. Heat-shock the cells for 90 s at 42 °C in a preheated water bath and place the cells back on ice for 5 min.

d. Add 1 mL of prewarmed LB medium to each tube and incubate at 37 °C for 60 min at 750 rpm.

e. Plate 100 μL of the transformed bacteria on a 20 mL LB agar plate supplemented with chloramphenicol (34 μg/mL) and spread the resuspension throughout the plate.

f. Centrifuge at 7,200× g for 1 min at RT and remove most of the medium with a pipette, leaving 100 μL to resuspend the rest.

g. Pipette the resuspension onto a 20 mL LB agar plate supplemented with chloramphenicol (34 μg/mL) and spread the resuspension throughout the plate.

h. Wait until the plate has dried, next to the flame.

i. Incubate plates overnight at 37 °C.

j. Isolate colonies in chloramphenicol (34 μg/mL) and grow them overnight at 37 °C and 250 rpm on 10 mL of LB media with chloramphenicol (34 μg/mL) for plasmid extraction.

k. Isolate plasmid using GeneJET Plasmid Miniprep kit.

6. Linearize 1 µg of pCC1FOS-oriT by PCR amplification using primers 2 and 3 (Table S1). See thermocycling conditions in Table 1.

**Table 1.** Thermocycling conditions for Q5^®^ high-fidelity DNA polymerase

Cycle step	Temperature	Time	Cycles
Initial denaturation	98 °C	10 min	1
Denaturation	98 °C	20 s	30
Annealing	50–72 °C (adjusted according to the melting temperature of primers)	20 s	
Extension	72 °C	20–30 s/kb	
Final extension	72 °C	2–10 min (adjusted to size of amplicon)	1
Hold	10 °C	∞	-

7. Amplify the CEN/ARS sequence from the plasmid pRS314 (Table S2) using primers 4–5 (Table S1). This genetic element is necessary to maintain the plasmid in yeast cells. See thermocycling conditions in Table 1.

8. Amplify the gentamicin resistance gene (*GmR*) from a pSEVA62 backbone using primers 6–7 (Table S1). See thermocycling conditions in Table 1.

9. Incorporate the Lox66 site through PCR amplification during an overlap PCR fusing CEN/ARS elements and the *GmR* gene using primers 4 and 7 (Table S1). See thermocycling conditions in Table 1.

10. Incorporate the CEN/ARS-GmR-lox66 in the vector pSB1C3 with Golden Gate assembly.

a. Dilute the target DNA fragment according to its size and concentration.

b. Add 2 μL of assembly mix, 1 μL of linearized vector, 1 μL of diluted DNA fragment (insert), and water up to 8 μL.

c. Set thermocycling conditions according to Table 2.

11. Linearize 300 ng of CEN/ARS-GmR-lox66 by PCR amplification for TAR cloning using primers 4–7 (Table S1). See thermocycling conditions in Table 1.

12. Incorporate the Loxm2/71 by amplification of the LEU2 using primers 8–9 (Table S1) from the plasmid pRS315 (Table S2). See thermocycling conditions in Table 1.

13. Amplify the Cre recombinase from the iGEM Part:BBa_K112122 (http://parts.igem.org/Main_Page) using primers 10–11 (Table S1). See thermocycling conditions in Table 1.

14. Assemble CRE-LEU2-loxm2/71 by overlap PCR using primers 12–15 (Table S1). See thermocycling conditions in Table 1.

15. Incorporate CRE-LEU2-lox2/71 in the vector pSB1C3 with Golden Gate assembly using primers 8–11 (to linearize vector). See thermocycling conditions in Table 1.

a. Dilute the target DNA fragment according to its size and concentration.

b. Add 2 μL of assembly mix, 1 μL of linearized vector, 1 μL of diluted DNA fragment (insert), and water up to 8 μL.

c. Set thermocycling conditions according to Table 2.

**Table 2.** Thermocycling conditions for Golden Gate assembly

Temperature	Time	Cycles
37 °C	20 min	1
16 °C	4 min	30
37 °C	3 min	
50 °C	10 min	1
80 °C	10 min	1

16. Linearize 300 ng of CRE-LEU2-lox2/71 by PCR amplification for TAR cloning using primers 12 and 13. See thermocycling conditions in Table 1.

17. Linearize 300 ng of each of the five fragments forming the lanthipeptide BGC by PCR amplification using primers 16–25 (Table S1). See thermocycling conditions in Table 1.

**Figure 2.**
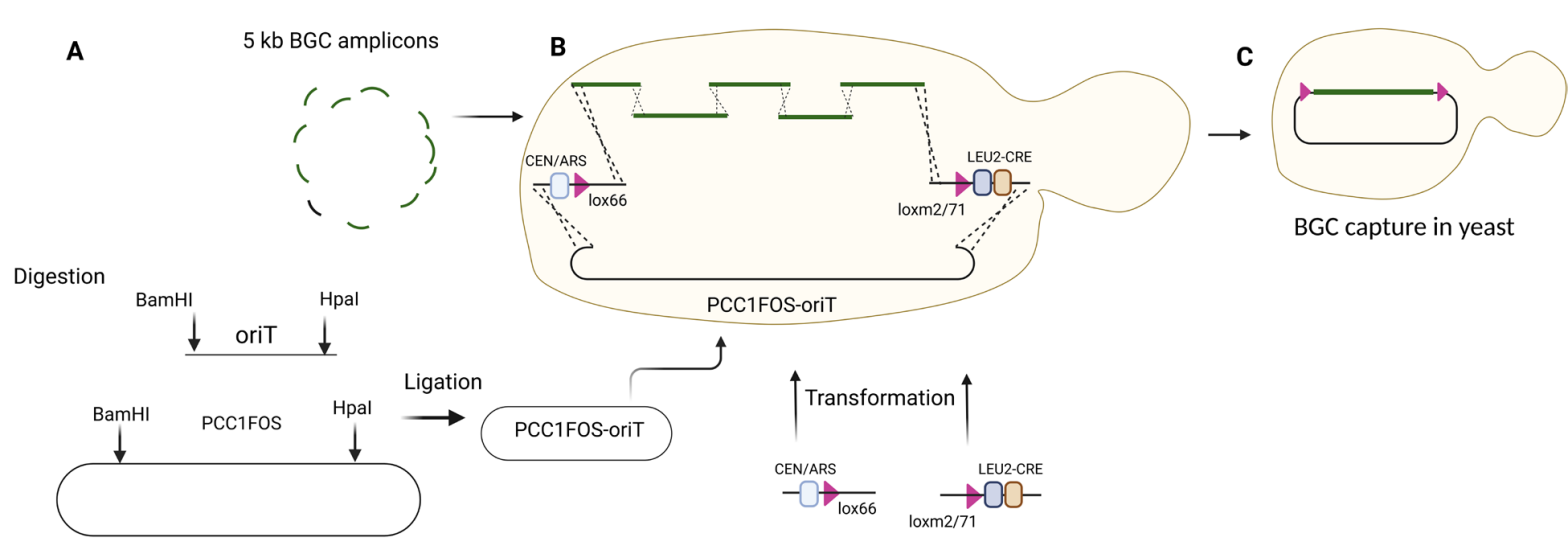
TAR cloning in yeast using 5 kb BGC amplicons, CEN/ARS-GmR-lox66, pCC1FOS-oriT, and CRE-LEU2-loxm2/71. (A) PCR amplification, digestion, and ligation of PCC1FOS and oriT. (B) Transformation of assembled PCC1FOS-oriT and 5 kb BGC amplicons in yeast. (C) BGC capture in yeast.

*Note: Design each cassette, amplicon, and linearized plasmid to contain 60-bp overhangs with the previous and following DNA fragment, enabling the capture of the lanthipeptide BGC, CEN/ARS-GmR-lox66, CRE-LEU2-lox2/71, and pCC1FOS-oriT in one step using transformation-associated recombination (TAR) cloning in yeast (Figure 2).*

**F. Transformation in yeast**

1. Day 1: Start yeast pre-culture. Start yeast cultures (50 μL from glycerol stock) in 5 mL of the appropriate medium (e.g., YPDA, min SD-LEU).

2. Incubate overnight at 30 °C and 190 rpm (starting around 6 pm). Autoclave enough 500 mL Erlenmeyer flasks for day 2.

3. Day 2: Measure the OD_600 nm_ (dilution 1/10). Measure the OD_600 nm_ in the morning (~9:30 am) and dilute accordingly in 5 mL of the appropriate medium, aiming at OD_600 nm_ = 2 by 6 pm. Check OD_600 nm_ again at ~2 pm and 6 pm.

4. Dilute accordingly, aiming at OD_600 nm_ = 2 the next day at 4 pm, keeping in mind a generation time of 90 min.

*Note: The calculation starts with an OD_600 nm_ =2 at 4 pm and then OD_600 nm_ =1 at 2:30 pm. Following this generation time, an OD_600 nm_ =0.000061035 is reached at 5:30 pm.*

Calculate the dilution using the following formula:

$${C_{1}v}_{1}=C_{2}v_{2}$$

Where C_1_ is the measured OD_600 nm_ on day 2, v_1_ is the initial volume to be calculated for the dilution, C_2_ is the final OD_600 nm_ = 0.000061035, and v_2_ is the final volume of 100 mL.

Yeast culture 1:

$$v_{1}\;\frac{\left(0.000061035)(100\;ml\right)}{C_{1}}$$

*Note: Adjust C_2_ according to the calculations with the generation time.*

5. Inoculate 2 × 100 mL of yeast culture with appropriate medium (use 500 mL Erlenmeyer flask) with the calculated volume in microliters.

6. Incubate at 30 °C at 190 rpm (until around 4 pm).

7. Day 3: Measure the OD_600 nm_ (dilution 1/10).

*Note: The expected OD_600 nm_ is between 2.6 and 2.8.*

8. Collect the cells by centrifugation at 1,892× *g* for 3 min at RT.

9. Resuspend each culture in 20 mL of sorbitol 1 M.

10. Divide each culture into 2× 50 mL tubes to centrifuge.

11. Resuspend pellet in 20 mL of sorbitol.

12. Transfer to the other tube to resuspend the other pellet. Store at 4 °C overnight.

13. Day 4: Yeast transformation. Before starting, prepare the following:

a. Prepare the PEG 20% and top agar (top agar can be prepared in advance and kept at 65 °C).

b. Refrigerate the centrifuge at 4 °C 15 min before centrifugation.

c. Put the sorbitol plates at 30 °C.

d. Prepare ~10 Spectro cuvettes with 1.35 mL of sorbitol 1 M and ~10 with 1.35 mL of SDS 2%.

e. Prepare two 50 mL Falcons, each with 40 mL of sorbitol 1 M.

f. Switch on the OD meter at 600 nm and blank with sorbitol 1 M.

14. Collect the yeast cells by centrifugation at 1,892× *g* for 3 min at RT.

15. Resuspend the pellet in 10 mL of SPEM.

16. Add 20 μL of β-Mercaptoethanol and 20 μL of Zymolyase-100T into the pellet from step F15 inside the fume hood. Keep the tube with sorbitol close.

17. Incubate in your hands for 5 min with mild agitation.

**CRITICAL STEP:** Overincubation can lead to a reduced number of correct transformants or no transformants at all due to excessive spheroplast lysis.

18. Verify the OD_600 nm_ of the following two sample preparations (A and B):

19. Sample A: With 150 μL of culture + 1.35 mL of sorbitol 1 M (1/10).

20. Sample B: With 150 μL of culture + 1.35 mL of SDS 2% (1/10).

*Note: The ratio of OD_600 nm_ between A and B should be 3–4 (normally around A = 0.8 and B = 0.2). Measure at 5, 7, 9, and 11 min and so on (every 2 min). Take samples as indicated until reaching the desired values.*

**CRITICAL STEP**: Ratios beyond 4 can lead to a reduced number of correct transformants or no transformants at all due to excessive yeast cell damage.

21. Quickly add 40 mL of sorbitol 1 M, mix by inversion, and recover the cells by centrifugation at 1,314× *g* for 5 min at 4 °C.

**CRITICAL STEP**: Not adding the sorbitol on time can lead to a reduced number of transformants or no transformants at all due to excessive spheroplast lysis.

22. Discard the supernatant and resuspend the pellet carefully in 20 mL of sorbitol 1 M using a 25 mL pipette.

23. Add 30 mL of sorbitol 1 M, mix by inversion, and centrifuge at 1,314× *g* for 5 min at 4 °C.

24. Discard the supernatant and remove the last drops with a pipette.

25. Resuspend carefully in 1 mL of STC: mix carefully by rotating the tube. Keep for 10 min at RT. Meanwhile, aliquot the DNA (use pipette tips with their ends cut off when transferring gDNA, since regular pipette tips can shear it mechanically when aspirating). You may keep the spheroplast for 1 h at RT at this point.

26. Aliquot the following four linearized DNA fragments into a 1.5 mL tube:

a. 1 μg of PCR-linearized pCC1FOS-oriT.

b. 300 ng of the linearized CEN/ARS-GmR-lox66 cassette.

c. 300 ng of linearized CRE-LEU2-loxm2/71 cassette.

d. 300 ng of each lanthipeptide BGC amplicon.

27. Add 100 μL of spheroplasts to each tube, mix by gently finger flicking the tube, and incubate for 10 min at RT.

28. Add 0.5 mL of PEG 20%, mix by inversion (4× strongly), and incubate for 20 min at RT.

29. Centrifuge the spheroplasts at 4,643× *g* for 5 min at RT.

30. Resuspend the pellets with 700 μL of SOS (mix gently with the pipette).

31. Incubate for 2 h at 30 °C. Put the TOP agar in the water bath at 55 °C.

32. Put 700 μL of spheroplasts of the transformation in a 15 mL tube and add 12 mL of TOP agar-leu agar.

33. Mix immediately by inversion (4–5 times gently) and plate. Put back the TOP agar-leu at 55 °C. Wait until the plates are solid and incubate at 30 °C.

34. The transformant colonies are normally seen after 3 days, but they can be kept up to 5 days at 30 °C to facilitate picking the colonies.

*Note: Yeast colonies will grow on top of or within the TOP agar-leu overlay.*

35. Patch yeast transformants onto SD-leu plates. Incubate the plates at 30 °C for 1–2 days.

**G. Screening yeast transformants by colony PCR**

1. Prepare Phire^TM^ Hot Start II DNA polymerase master mixes with the pairs of primers in Table 3. The sequence of primers can be found in Table S1.

*Note: Given that colony PCR amplicons are expected to have different sizes, a multiplex PCR using multiple pairs of primers can be performed in one reaction.*

**Table 3.** Colony PCR primers and expected amplicon sizes of the lanthipeptide BGC assembly

Colony PCR amplicon	Expected size (kb)	Primers
1	0.6	26–27
2	0.7	28–29
3	1.5	32–33
4	1.7	34–35

2. Prepare individual colony PCR reactions of 20 μL in PCR tubes.

3. Pick *S. cerevisiae* W303 transformants with sterile tips and transfer them to PCR tubes with Phire master mix. Place each tip in a different PCR tube.

*Note: Barely touch yeast colonies, since taking excessive DNA template can inhibit the reaction or lead to nonspecific amplification.*

4. Discard tips after direct contact of the colony with each PCR reaction tube.

5. Run the PCR according to the thermocycler conditions in Table 4.

**Table 4.** Thermocycling conditions for colony PCR using Phire^TM^ Hot Start II DNA polymerase

Cycle step	Temperature	Time	Cycles
Initial denaturation	98 °C	10 min	1
Denaturation	98 °C	15 s	20
Annealing	50–72 °C (adjusted according to the melting temperature of primers)	15 s	
Extension	72°C	10 s/kb	
Final extension	72°C	1 min	1
Hold	10 °C	∞	1

6. Prepare 100 mL of 1% agarose gel in 1× TAE buffer with 10 μL of MIDORI Green Easy in a wide mini-sub cell casting tray with 2 × 26-well combs.

7. Place the agarose gel in the wide mini-sub cell apparatus and add enough 1× TAE to cover the gel. Load the first well of each comb with 4 μL of 1 kb Plus DNA ladder.

8. Add 4 μL of 6× DNA loading dye to each PCR reaction.

9. Add 10 μL of each PCR reaction starting from well number 2.

*Note: Loading gels with 26-well combs can be facilitated using multi-channel pipettes.*

**Figure 3.**
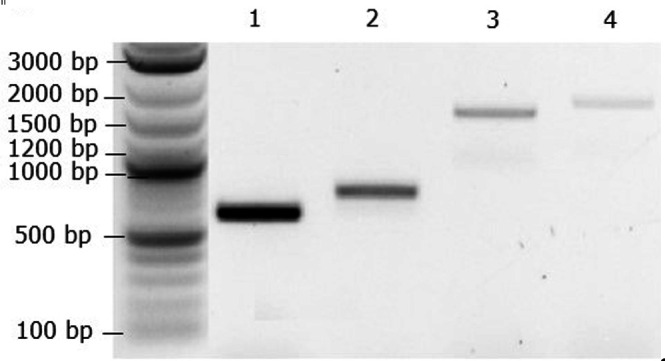
Gel electrophoresis with amplicons 1, 2, 3, and 4, as shown in Table 3

10. Run the gel at 100 V (constant) for 30 min using a PowerPac Basic Power Supply (Mupid-One Electrophoresis Unit).

11. Visualize the gel in a gel doc XR. Follow the manufacturer’s instructions for visualization.

12. Identify positive transformants with amplicons of sizes shown in Table 3 and Figure 3.

13. Grow positive colonies of *S. cerevisiae* W303 overnight at 30 °C and 250 rpm in 10 mL of SD minimal media depleted of leucine. This is the same media as in sorb plates, but without agar.

**H. Isolation of total yeast DNA and transformation into TransforMax^TM^ EPI300^TM^ *E. coli* cells**

1. Extract total DNA using the Yeast DNA Extraction kit. Follow the manufacturer’s instructions.

2. Pipette the chilled DNA/bacteria solution into the chilled 0.2 cm gap-width cuvette. Follow the manufacturer’s instructions.

3. Electroporate *E. coli* cells in a gene pulser electroporation system with the following settings: 2.5 kV, 25 μF, 200 Ω.

4. Immediately following the electroporation, add 1 mL of LB liquid media for recovery.

5. Transfer the electroporated bacteria in LB into a 1.5 mL microcentrifuge tube.

6. Incubate the 1.5 mL microcentrifuge tube at 37 °C for 1 h with shaking at 250 rpm.

7. Plate 100 μL of the electroporated bacteria on an LB agar plate containing 15 μg/mL gentamycin.

8. Centrifuge at 7,200× *g* for 1 min at RT, remove most of the medium with a pipette, resuspend the rest, and spread the resuspension throughout the plate containing 15 μg/mL gentamycin.

9. Incubate the plate at 37 °C for 1–2 days.

10. Grow positive colonies of *E. coli* cells overnight at 37 °C and 250 rpm in 10 mL of LB media with gentamycin (15 μg/mL).

11. Isolate plasmid using GeneJET Plasmid Miniprep kit. Follow the manufacturer’s instructions for plasmid isolation.

12. Verify the sequence of isolated plasmids from *E. coli* EPI300 using whole plasmid sequencing (Plasmidsaurus inc.).

## Data analysis

The quality and completeness of the assembled genome were evaluated using CheckM [24] and BUSCO [25]. Assemblies with completeness <90% are typically considered incomplete; therefore, additional polishing or a hybrid assembly with short reads would be required to achieve a high-quality genome.

## Validation of protocol

This protocol has been used and validated in the following research article:

- Campos-Magaña et al. [18]. Enabling Access to Novel Bacterial Biosynthetic Potential From ONT Draft Genomic Data. *Microbial Biotechnology.* Figure 3 and Supplementary Figure 6.

After extracting the shuttle vector from *S. cerevisiae* W303 and amplifying it in *E. coli* EPI300 cells, we demonstrated that the complete BGC was assembled as intended by whole plasmid sequencing using ONT (Plasmidsaurus inc.). Furthermore, we performed Sanger sequencing of the entire gene cluster assembled in the vector. The alignment of Sanger sequencing with the original ONT sequencing showed 26 mismatches and 93 gaps/insertions from a total of 26 kb (99.5% identity) as can be observed in Figure S6 in [18], demonstrating that ONT sequencing has achieved high levels of accuracy. To ensure that these mismatches are not mutations generated during the PCR amplification and assembly process, we also performed Sanger sequencing on the genomic DNA, and only two mismatches were found, all producing silent mutations. In all, the sequencing precision given by ONT allowed obtaining sufficient data to accurately assemble the whole gene cluster in a plasmid vector. The Sanger polished sequence of the BGC is available at ENA accession PRJEB72099.

## General notes and troubleshooting

**General notes**

1. As in any cloning method, the transformation efficiency will decrease as the number of fragments to be assembled increases or as the size of the target BGC increases. The transformation efficiency will also decrease when DNA is too complex, such as in high GC content and/or highly repetitive regions within BGCs.

3. The sizes of the BGC amplicons to be assembled were intended to be as similar as possible among all; in this study, all BGC fragments were approximately 5 kb. However, shorter or larger size fragments could be used.

3. Although we only tested a concentration of 300 ng of each fragment to be assembled, it is possible to test other concentrations during TAR cloning in yeast, depending on availability. If DNA amounts allow, performing increasing molar ratios of fragments to pCCIFOS-oriT may help in optimizing the assembly reactions.

4. Although we used 60 bp overlaps in each fragment to be assembled, longer homologous overlaps can improve correct recombination frequency.

**Troubleshooting**

Problem 1: The 3–4 ratio of OD600 nm between A and B is not reached because there is no conversion to spheroplasts.

Possible cause: Insufficient zymolyase activity.

Solution: Incubate the reaction longer until the desired results are achieved.

Problem 2: Lysed spheroplasts.

Possible cause: Spheroplast are weakened cells, and overincubation can lead to lysis. The concentration of zymolyase was very high, or shaking was excessive for the cells.

Solution: Shorten the incubation time or reduce the concentration of zymolyase used.
